# Fallout: the psychosocial harms of negative military discharge experiences

**DOI:** 10.3389/fpsyg.2025.1521056

**Published:** 2025-05-08

**Authors:** Cameron Grant, Lydia Woodyatt, Henry Bowen, Jonathan Lane

**Affiliations:** ^1^College of Education Psychology and Social Work, Flinders University, Adelaide, SA, Australia; ^2^Flinders Institute of Mental Health and Wellbeing, Open Door, Adelaide, SA, Australia; ^3^Military and Emergency Services Health Australia, Adelaide, SA, Australia; ^4^School of Medicine, University of Tasmania, Hobart, TAS, Australia

**Keywords:** veteran, military, transition, identity, institutional betrayal

## Abstract

Military discharge marks a pivotal life transition, often resulting in loss of identity, purpose, and belonging. Negative discharge experiences are further associated with feelings of institutional betrayal. This study explored which aspects of discharge negatively impact veterans during and after their transition. Using Reflexive Thematic Analysis on accounts from Australian veterans (*N* = 313), three key themes emerged: (1) *Discharge Experiences as Institutional Transgressions and Betrayal*, with sub-themes: *Unceremonious Exits and Lingering Discharges*, *Left Harmed and Rejected*, and *Bad Actors and Acutely Harmful Events*; (2) *Discharge as a Loss of Self*; and (3) *Negative Discharge Experiences as Negative Centralizing Events and ‘Stuck-Points’*. Findings revealed that veterans experience harm when they perceive their discharge as an institutional or personal transgression—ranging from bureaucratic disregard to service-related injuries leading to rejection and overt betrayal by bad actors. These events, regardless of severity, undermine veterans’ shared military identity and values, posing a psychological threat to their sense of belonging, severing familial-like bonds, and fostering feelings of rejection, diminished self-worth, isolation, and betrayal—hindering transition and identity reconstruction. We argue that the harm stems not from discharge itself but from veterans experiencing these negative experiences as a violation of shared values—values they were required to embody for group membership. In identity-centric workplaces like the military, where enculturation fosters deep, family-like bonds, discharge represents a unique psychosocial hazard. Proactive management is essential to mitigate lasting psychological harm.

## Introduction

Military discharge represents a pivotal moment in the lives of service members, marking the end of their military careers and the beginning of their reintegration into civilian life (Romaniuk et al., 2018). However, this transition is often accompanied by a range of challenges, particularly for those who are involuntarily or medically discharged. These individuals face significantly higher risks of adverse mental health outcomes, suicidality, and difficulties with transition and reintegration compared to their generally discharged peers (Sadler et al., 2021). The unique challenges faced by specific cohorts highlight the importance of the discharge experience itself in creating vulnerability. Therefore, the nature of the discharge experience, irrespective of the type of discharge, may be a key determinant of mental health outcomes and successful reintegration into civilian life.

Yet, there is a considerable gap in our understanding of how various discharge experiences, regardless of discharge type, impact the transition process, long-term mental health outcomes, and reintegration. While it is well known that discharge can lead to feelings of isolation, rejection, and identity loss, the specific experiences and factors precipitating these feelings and how they hinder successful transition remain unclear (Romaniuk et al., 2018). Indeed, previous quantitative research (Grant et al., 2025) has shown a correlational relationship between negative discharge experiences, feelings of institutional betrayal, identity loss and poorer wellbeing and mental health outcomes years after discharge. In this research we adopted a qualitative approach to investigate the discharge experiences of veterans, why discharge experiences might be related to both institutional betrayal and poorer wellbeing, in order to understand how we can better support service personnel in their transition out of military life. Identifying and understanding these factors is crucial to enhancing the military discharge experience and developing support systems and interventions that mitigate negative outcomes and promote healthier transitions for all service members.

### Military discharge experiences and institutional betrayal

Recent testimonials from the Royal Commission into Defence and Veteran Suicide (2024a) indicated that institutional betrayal may peak after discharge, with inappropriate support during the transition process being a critical factor in perceptions of betrayal. Institutional betrayal is defined as harm caused by an institution to its members or dependents through actions or inactions, such as failing to prevent misconduct, providing inadequate responses to wrongdoing, or creating an environment that facilitates harm (Smith and Freyd, 2014). Military populations may be particularly susceptible to institutional betrayal due to the unique culture and structure of the military, which demands strict conformity, rigorous training, and adherence to a clear hierarchy with significant power imbalances (Holliday and Monteith, 2019; Monteith et al., 2021). Service members often suppress or redefine their personal identities to adopt a collective military identity, leading to deep reliance on the institution to fulfil psychological and social needs in addition to practical needs, such as financial security, healthcare, and career advancement (Flack and Kite, 2021; Holliday and Monteith, 2019; Lane and Wallace, 2020; Monteith et al., 2021). This extensive dependency heightens the risk of experiencing institutional betrayal and amplifies the harm when such betrayal occurs, impacting multiple critical aspects of their lives simultaneously (Smith and Freyd, 2014).

Research on institutional betrayal within the military has predominantly focused on instances of military sexual assault but may be an important factor in military discharge as well. Studies have found that survivors of military sexual assault face significant barriers when reporting assaults, including disbelief, inadequate responses or punitive actions against them rather than the perpetrators (Holliday and Monteith, 2019; Monteith et al., 2016). These inadequate and harmful responses, in addition to the assault itself, give rise to feelings of isolation and an erosion of trust and safety within the institution (Holliday and Monteith, 2019). Consequently, this assault itself and the inappropriate responses can both be perceived as betrayals. These institutional betrayals are in turn associated with PTSD symptoms and/or exacerbated PTSD symptoms, increased mental health difficulties and suicide risk (Christl et al., 2024; Monteith et al., 2016).

Given veterans face increased risk and vulnerability to institutional betrayal and its significant impact on mental health and well-being, it is important to consider how institutional betrayal may also manifest in other critical areas of military life, such as during the military discharge process. The transition from the military is a known vulnerable period for members, marked by significant losses of identity, culture, purpose and reintegration difficulties (Romaniuk et al., 2018). However, if members perceive that the military has harmed them or failed to adequately support them during this critical transition, this may contribute to feelings of betrayal. Indeed, recent testimonials from the Royal Commission into Defence and Veteran Suicide (2024a, pp 31–43) link negative discharge experiences, institutional betrayal, and negative mental health consequences. Further recent research demonstrates that a perceived negative discharge experience is associated with institutional betrayal, identity loss, and poorer mental health and well-being (Grant et al., 2025).

### Overview

Recent quantitative research has identified discharge experiences as significant factors in veterans’ identity transitions and mental health (Wadham et al., 2023). However, quantitative approaches provide limited insight into the complexity of these experiences. The qualitative approach of this study allows the investigation into the nuanced experiences of military discharge through a detailed thematic analysis of veterans’ self-reported experiences of military discharge. By centering veterans’ narratives, we aim to examine how discharge experiences shape veterans’ identity transitions and psychological outcomes, with particular focus on institutional practices, cultural dynamics, and individual interpretations.

The primary research questions were;

1. What specific aspects of military discharge experiences contribute to negative transition outcomes for veterans?
2. How do veterans perceive and make meaning of negative discharge experiences, particularly in relation to their identity, belonging, and sense of institutional betrayal?
3. What are the mechanisms through which negative discharge experiences might impact veterans’ wellbeing and mental health in the long term?

The generated themes illustrate the complex interplay of interpersonal and institutional dynamics, including feelings of rejection, disrespect, devaluation, and institutional transgressions ranging from neglect to intentional harm. These perceived violations of trust and justice significantly impact the quality of the discharge process. For some veterans, the discharge event becomes a negative central experience that irrevocably alters their life trajectory. Respondents emphasize the importance of these dynamics in shaping their perceptions of their service’s worth, their inherent value post-service, and their sense of belonging, all of which have enduring effects on identity, self-worth, and the ability to reintegrate into civilian life.

## Methods

### Theoretical foundations

Our research adopted a subtle realist perspective, asserting that there is an objective reality, but individuals’ understanding of that reality is constructed through individual subjective cognition, influenced by personal experiences, perceptions and social constructs (Hammersley, 2018). This approach values participants’ experiences and conceptualizations as legitimate reflections of their social reality, acknowledging that their responses may be constructed via various social, cultural, historical, and theoretical influences. By adopting this perspective, we aim to deeply understand and interpret the nuanced experiences and narratives provided by our participants.

While we recognize that interactive methods such as interviews would allow for probing, clarification, and analysis of non-verbal cues, our choice of open-text survey responses enabled us to collect a larger and more diverse sample across Australia, including veterans spanning nearly six decades of service. This approach facilitated the inclusion of perspectives that might otherwise be difficult to access through interviews, particularly given the sensitive nature of discharge experiences and the geographic dispersion of veterans. The richness and depth of the voluntary narrative responses (ranging up to 384 words per response) provided substantial material for meaningful analysis. Nevertheless, we acknowledge this methodological limitation and its potential impact on the depth of certain individual narratives in our Discussion section.

We employed reflexive thematic analysis, which is well-suited for this study due to its acknowledgment of the subjective and socially constructed nature of experiences and its emphasis on the researchers’ role in interpreting the data (Braun and Clarke, 2021). This approach enabled us to engage deeply with the data, allowing for a comprehensive exploration of the complexities of Australian military veterans’ discharge experiences.

### Researcher background

#### Reflexivity

Reflexivity is vital for ensuring empirical rigor, transparency, and validity in qualitative research (Darawsheh, 2014; Probst and Berenson, 2014). This critical methodological practice involves continuous, explicit self-awareness and critical self-reflection by researchers on their potential biases, preconceptions, and relationship to the research topic, participants, and analytical process. During this study, we embraced reflexivity by frequently reflecting on how our identities as researchers, military veterans, and mental health clinicians impacted the research process and interpretation of the data. The principal investigator of this project (Grant) is an Australian Army veteran who served at the rank of Private as an Infantryman and then as a Supply Operator for six years total before a medical discharge in 2015. He has worked as a lived-experience peer worker while completing an undergraduate honors degree in psychology and is currently a final year Clinical Psychology PhD student and registered psychologist. Each of these experiences shaped his reading and interpretation of the accounts provided. To incorporate a diverse perspective, the coauthor team includes a civilian social psychologist who specialises in justice, transgressions and moral repair (Woodyatt), a civilian qualitative mental health researcher focussed on the experiences of military and emergency service workers (Bowen), and an Australian Army veteran and Psychiatrist (Lane).

While our goal as a team was to give voice to the experiences of the veterans who took the time to tell us their stories, we acknowledge that we are always translating and interpreting, and our subjectivity is a part of that activity. Nonetheless, the project was led by a lived experience researcher, who shares some cultural and experiential commonalities with the participants. This enhanced our team’s understanding and contextualization of the participant’s responses and guided the thematic analysis process. Ongoing discussions between the first 3 authors throughout the thematic analysis process led to the critical appraisal and development of themes that were transparently and rigorously derived from the data. The fourth author contributed to validating the findings based on his clinical and personal experiences.

### Participants

To be eligible, participants had to have served one day or more of continuous full-time service in the Australian military and no longer be serving in the Australian military in any capacity. In total, 379 participants fully completed the survey. Collectively, 313 (82.6%) of participants provided at least one qualitative response ranging from one-word responses to detailed 384-word responses, with a mean length of 80 words of responses per participant per question. Participants were Australian military veterans who were predominately non-commissioned (other ranks *n* = 124 non-commissioned *n* = 209, and commissioned *n* = 46), older (*M* = 57 years, *SD =* 13.1, range 25–81 years), and male veterans (male *n* = 332, female *n* = 46, did not describe *n* = 1). Veterans served in the Army (*n* = 209), Air Force (*n* = 97), Navy (*n* = 97), one participant preferred not to say (*n* = 1). Most veterans had not been operationally deployed (*n* = 233) during their length of service (*M* = 14.7, *SD* = 10.3 years) before discharging (general discharge *n* = 220, medical discharge *n* = 108, retirement *n* = 32 and administrative discharge *n* = 19). There was a substantial difference in time since discharge among participants (*M* = 23.40, *SD* = 14.90, range 0 to 58 years).

### Ethics

This study was approved by the Flinders University Human Research Ethics Committee (Project Number HEL4980). All relevant information regarding the study and the right to withdraw was provided to the participants before they digitally consented to participate in the study. Participants were de-identified, and support hotlines and service information were provided in case of discomfort or distress.

### Procedure

Data from this study was extracted from a larger quantitative study (Grant et al., 2025), which recruited participants using community snowball sampling online initiated via targeted advertising on Facebook and Instagram. The advertisement invited veterans to participate in a survey on military discharge experiences, identity, and mental health. Eligible participants had to have served in the Australian military and no longer be serving. Participation was incentivized by a $5 donation to a veteran charity of choice.

The richness of participants’ voluntary open-text responses exceeded our initial expectations, providing unexpectedly valuable insights that warranted dedicated qualitative analysis. While this voluntary open-text response format precluded probing and clarification typical of qualitative research, this approach enabled us to analyze perspectives from a diverse sample spanning nearly six decades of service across Australian veterans. Participants voluntarily (non-forced response) provided qualitative data in response to the below prompts.

1. *Your experiences are valuable. If there is anything you would like to add about your discharge experience or transitioning back to civilian life, please share below.*
2. *If there is anything you would like to add about your experiences in relation to Military discharge and your sense of self, identity, or belonging, the transition to civilian life and mental health and well-being, please do so below.*

Question 1 was asked after an identity loss scale and discharge-related institutional betrayal questionnaire. Question 2 was asked at the end of the survey, post mental health and wellbeing measures and demographic questions. Basic spellchecking was completed on some of the quotes and acronyms were broken down to aid legibility.

Participant demographic information (age, gender, years served, and discharge year) is included with each excerpt to provide important context and create a more complete impression of veterans’ experiences across different service periods, career lengths, and time since discharge. This contextual information helps illustrate how discharge experiences may vary or show consistency across different demographic profiles and military backgrounds.

## Results

### Data analysis

We conducted reflexive thematic analysis following Braun and Clarke (2021) “Thematic Analysis: A Practical Guide.” NVivo v.11,© QSR International was utilized to manage the data and develop a code book and subsequent themes. Two researchers independently coded the data through an iterative process. The process involved data familiarization through thoroughly reading and re-reading the data and noting initial observations, followed by generating codes to capture the common and important features within the transcripts. The entire dataset was initially coded before initial themes were generated by examining the codes and collating data to identify broader patterns of meaning. The process of theme searching, generation, and defining was iterative.

The team developed preliminary themes through intensive discussions, continuously revisiting original data to ensure authenticity. Our multidisciplinary team composition—including both veterans (Grant, Lane) and non-veterans (Woodyatt, Bowen)—enabled triangulation of perspectives, enhancing analytical depth while acknowledging that interpretation is inherently influenced by researchers’ backgrounds and perspectives. We moved from descriptive organization to interpretative analysis, identifying conceptual relationships that illuminated how institutional practices, cultural dynamics, and interpersonal relationships shaped veterans’ experiences of military discharge.

### Verification strategies

We strengthened analytical rigor by ensuring responsiveness throughout the research process. We iteratively moved between research stages to maintain methodological coherence, continuously assessing sampling appropriateness and data sufficiency. We systematically searched for contradictory cases that challenged our developing themes, incorporating these perspectives to ensure our framework captured the complexity of veterans’ experiences. Our analysis employed thick description through extensive participant quotations, grounding interpretations directly in veterans’ accounts and allowing readers to evaluate our analytical claims against the raw data. Throughout analysis, we thought theoretically—connecting emerging patterns with existing frameworks while remaining open to novel conceptualizations that better captured veterans’ lived experiences.

### Theme 1 – Discharge experiences as institutional transgressions and betrayal

This theme primarily addresses research questions 1 and 3 by identifying specific negative aspects of military discharge and uncovering a key mechanism—the violation of shared values—through which discharge experiences can create harm. This theme also addresses research question 2 by revealing that some veterans’ perceive these experiences as betrayals and expectation violations.

Across many participants, it was clear that discharge experiences were viewed as a betrayal of shared values, norms and expectations they had about being a service person and a member of Defence. In this way, across many of the participants, we could see evidence of feelings of betrayal, having been transgressed against by the organization, and in some cases, these feelings resulted in rumination, anger, and bitterness similar to what we would expect when someone has experienced (and not resolved) a transgression. While participants were not often explicit in their expectations of what should occur in discharge, it was very evident that these implicit expectations were not met. We observed this theme in three expressions which we identified as subthemes.

Sub-theme 1, ‘*Unceremonious exits and lingering discharges*’ highlights how the lack of basic recognition and support leads to perceived harm. Sub-theme 2, ‘*Harmed and rejected*,’ captures veterans’ feelings of rejection following involuntary or medical discharge, often due to service-related injuries. Lastly, sub-theme 3 ‘*Bad actors and acutely harmful events*’ involves intentional transgressions by individuals who abused power, insulted, or inflicted harm beyond normal service expectations or requirements.

### Sub-theme 1: Unceremonious exits and lingering discharges

Many veterans reported an unceremonious exit, encompassing experiences of a passive and subtle sense of rejection. While generally not involving explicit transgressive acts, many participants spoke about a complete lack of process or support for leaving, an absence of any acknowledgement of service, or actions that they experienced as violations of basic shared military values. Several participants reported the absence of appropriate recognition of their service and rank during or that their discharge charge processes were completed by people of inappropriate rank (including civilians). For example, *Participant* 21 (age 73, served 23 years, discharged 1993) *“Person behind counter did not respect my rank. He was a corporal; I was a WO2”.* Respecting rank is a core value of the military. It acknowledges the person’s authority, experience, dedication, and time in service to achieve that rank and them as superior within the chain of command and institution. Therefore, when Participant 21’s rank was not acknowledged, this transgression represented a values violation that was disrespectful and, in the context of his last day of service, remains salient even 23 years after his discharge.

Many veterans reported a discharge characterized by feelings of a rushed discharge process and lack of preparation. These veterans reported having no transition experience at all and a general sense of institutional indifference to their discharge. One member who was on leave at the time of their discharge reported the trivial process, with no contact from the institution other than letter correspondence.

*“There was none [a transition], watch that the door does not hit your arse on the way out […] One medical exam later, a few pieces of paper shuffled and that was it, not even a pamphlet or any advice, I was just… gone. It was a pitiful experience with no contact apart from a letter or two due to being on leave at the time.” Par*ticipant 89 (Male aged 58 years, served 12 years, discharged in 2001).

One veteran noted the stark contrast between recruitment processes, fully supported by the military, and discharge processes, which are self-directed to minimize administration.

*“There just wasn’t an easy-to-understand discharge process. The onus was on the individual to seek out the appropriate forms and process and get all the paperwork signed off by the relevant sub-units. Very different to enlisting when you were spoon-fed every step of the way as a group. I feel like this is by design rather than accidental since it would be easier to stay in from an admin perspective.”* Participant 96 (Male, aged 33, served 4 years, discharged 2014).

Even when efforts were made to farewell members, these were done inconsistently and at times completed by people who were part of the reason that the person was leaving the military. One veteran described his farewell presentation as a lackluster and insulting event due to his command’s obstructive attitude toward his discharge process preceding the event. *“On my final day, I was humiliated in front of the unit and my wife with a half-arsed presentation from the Unit’s Command.”* Participant 40 (Male aged 57, served 37 years, discharged 2022). This suggests that ‘going through the motions’ after instances of perceived harm can make such gestures seem disingenuous and perpetrate further harm, highlighting the need for consistency of support throughout the discharge process.

For some, negative feelings associated with these events persisted long after discharge, especially those who viewed their service as having high personal costs or those who served for an extended period. For them, this ‘unceremonious exit’ represented a more profound transgression that affected them long after service. *“[…] that was the lack of respect shown…it still annoys me and it was 30 years ago”*. Participant 46 (Male aged 58, served 11 years, discharged 1993).

Many veterans tended to interpret these ‘unceremonious exits’ not as systematic failures experienced by everyone but as personalized isolated events, potentially amplifying their lingering impact. For example, Participant 259 (male, age 56, served 24 years, discharged in 2012) personally facilitated and valued recognizing and farewelling others but did not receive a farewell himself. This omission left him feeling deeply betrayed with a negative view and regretful attitude toward his service, which had long-lasting negative effects on his sense of self and belonging in both military and civilian contexts, with the memory of the event troubling him long after discharge.

*“In the last 5 years of my career I organised section BBQs cake and farewell presents for people in my area who were leaving. On my last day there was nothing. So I left at lunchtime. No welfare check was ever made and I was feeling suicidal at the time. I felt used and betrayed after 24 years service. I’ve had nothing to do with the military since. I still cannot bring myself to go to ANZAC day […]. I regret my 24 years of service. I regret the damage that my time in service that is still affecting my life and family. I’m not in the military anymore but I’m still not comfortable to civilian live. I just do not fit in. I am broken.”* Participant 259 (male age 56, served 24 years, discharged 2012).

Many members reported feeling disconnected and lacking a sense of belonging well before their discharge date. They observed that once they submitted their discharge papers or were flagged for involuntary discharge, the military’s concern for their well-being diminished, and they were treated poorly, with indifference or as a nuisance. In some instances, veterans reported being segregated from their regular workplace and peers, creating or exacerbating a sense of isolation and abandonment. This was more common among members who reported a protracted discharge, such as those awaiting a medical review board to confirm medical discharge. They reported feeling physically ‘in’ but socially and psychologically ‘out’ of the military.

*“Once someone is discharging, they are treated like a second class citizen, even punished for inconveniencing the CoC [Chain of Command] because they have other life goals”* Participant 236 (male, aged 32, served 7 years, discharged 2022).

*“Chain of command does not care. No one bothers to follow up on your well-being whilst on medical leave pending discharge. I was a highly valued member one day then totally discarded and not worth any effort on the next day.”* Participant 258 (Female, aged 52, served 25 years, discharged 2022).

One participant described the negative consequences of a protracted discharge process while medically downgraded. *“My final 18 months in the Army was spent downgraded and I slowly felt myself becoming an anxious, miserable shell of the person I used to be.”* Participant 79 (Female, aged 33, served 6 years, discharged 2022).

Another participant reported receiving positive support from the transition cell but a general sense of disregard from her chain of command, which she perceived may have been justified. This experience suggests that disregard from one’s unit during discharge may be common or even anticipated.

*“During discharge, I felt well looked after by the teams responsible for the discharge process (IE Transition Cell) but my chain of command were difficult and did not farewell me. I had a strong sense that I was leaving the team so I no longer deserved their time, which is maybe true.”* Participant 295 (female, aged 33, served 11 years, discharged 2022).

This underscores the critical role of interpersonal context in shaping the transition experience—the “who” in support and positive interactions matters. In the quasi-familial military culture, leaders often serve as role models or parental figures, while peers are regarded as siblings (Meyer et al., 2015). Consequently, interactions with established peers and leadership within the unit are likely to have a more profound impact on a member’s sense of belonging and self-worth during discharge compared to interactions with a transition cell with which they have no established connection, even when the latter is supportive and respectful.

Transgressions occurring close to the time of military discharge are likely to elicit negative emotions and outcomes, increasing the likelihood that the discharge becomes a negative centralized event in the veteran’s narrative – an event that defines their sense of identity post-service and potentially hinders adaptive adjustment (Fitzgerald et al., 2016). Many veterans describe their negative discharge experiences in vivid detail, highlighting the lasting and ongoing repercussions on their sense of self, worth, belonging, and overall well-being long after the event.

Though many of the examples in this sub-theme are seemingly minor issues of procedural neglect, we argue they are significant because they violate the shared values that bind military members to each other and the institution and form their military identity, which is often the largest part of a member’s self-concept (Flack and Kite, 2021). Value violations can occur through action (e.g., purposefully making a member’s discharge difficult) or inaction (e.g., not acknowledging service or providing a farewell). Being victim to such transgressions affects our core psychological needs, particularly our need for belonging, or - more specifically, a sense of social-moral identity – a sense that we are good, respected, and appropriate group members or relationship partners, defined by the social groups with which we identify ourselves (Baumeister and Leary, 1995; Woodyatt et al., 2022). The need for social-moral identity (need for belonging) is the basis from which we derive our self-esteem, sense of worth, and ultimately shape our personal identity (Leary and Baumeister, 2000; Leary et al., 2015). Consequently, transgressions from our in-group represent value violations that disrupt our sense of social-moral identity, challenge our self-concept as ‘good ‘and valued members, and can signal social exclusion, leading to psychological self-uncertainty (Woodyatt, 2023).

Values are the foundation of military group membership and are aggressively imposed through deliberate enculturation processes during enlistment and reinforced throughout service (Coll et al., 2011; Dabovich et al., 2019). They are reinforced formally by military law and informally by social norms, differentiating members from civilians and defining military group membership (Agostino, 1998). Therefore, we argue that seemingly minor transgressions can be perceived by *some* veterans, particularly when nearing military discharge (a period primed for identity loss and literal removal of group membership), as significant betrayals incongruent with the shared values that bind the member and the institution. This rupturing of values can be perceived as rejection and lead to a sense of alienation and uncertainty about oneself as a ‘good’ and valued group member. Given that people experiencing discharge from the military are going through a major period of identity transition (Flack and Kite, 2021; Grant et al., 2025; Haslam et al., 2021; Thompson et al., 2017) these psychological threats and needs are likely intensified, making such experiences more psychologically salient and likely to have enduring effects on their mental well-being.

### Sub-theme 2 – Left harmed and rejected

Participants in this sub-theme reported more overt transgressions and instances of harm or rejection compared to the more subtle and procedural neglect of sub-theme 1. This sub-theme was particularly visible in, but not isolated to, the accounts of people who were medically or involuntarily discharged.

Some veterans reported feeling overtly rejected after being medically or involuntarily discharged, interpreting their separation as an explicit rejection *“I was medically discharged after 19.5 years of service. I did not want to leave, but they did not want to keep me.”* Participant 110 (male, aged 44, served 20 years, discharged 2022). Often, these veterans would attribute blame to the military institution for overtraining them until physical or psychological injury, making them unfit for service and leading to what was perceived as an underserved and often sudden, cold and callous discharge with ongoing negative psychological effects and a reappraisal of their service. This reflects the concept of institutional betrayal where individuals were harmed firstly by an event(s) and then by the perceived inappropriate response, being forced to discharge.

*“You get overused until you break mentally then they throw you away like old rubbish.”* Participant 2 (Male aged 62, served 30 years, discharged 2009).

*“I was medically discharged after 17 years, in one day, out the next. No transition whatsoever; to this day, it still affects me.”* Participant 131 (Male, aged 67, served 17 years, discharged 1991).

*“Medically discharged after 32 years. Wasted 32 years of my life in the RAAF”* Participant 19 (male, aged 58, served 32 years, discharged 2013).

Here, we see a double rejection, first in the institution not living up to the implicit expectations that the military should care for its own and the feeling of being precluded from further service as a consequence of harm. The harm caused wasn’t simply the original events themselves (resulting in physical or psychological harm) but how this was then translated into a lack of care and support during the discharge process. Like the quote above from Participant 2, several participants used terms implying that they felt treated as less than human, like objects “overused” and “thrown away”, or in the examples below, like a “dirty arse” or “pawn.”

*“Felt I was wiped like a dirty arse. I use the phrase, The Army treats you like a prostitute, when your body is good it’s all go but when it fails out you go.”* Participant 247 (Male, aged 77, served 38 years, discharged 2002).

*“I feel the military treat you like a pawn. Program you and get what it needs then when your no worth to them anymore or you decide to separate they cut you off as if you never existed. Even when you are part of their grand plan and team your family and loved ones are treated like burdens that are a problem not a support to you and defence.”* Participant 142 (Male, 57, served 24 years, discharged 2005).

For one veteran, a final act of harm was perpetrated months after their discharge when a military member contacted her for a welfare check before commenting on her ‘bad attitude’, leading to a revaluation of the worth of her service and her perceived worth to the institution.

*“[…] so when I was told I had a bad attitude after so long, it made me realise that no one actually cared about me as a person. I was just a number to them, one they were just going to replace with the next posting cycle.”* Participant 90 (Female, aged 29, served 8 years, discharged 2022).

Blame for these experiences of harm was often assigned to individuals, their unit’s chain of command or administratively cumbersome and inefficient discharge processes. For example, Participant 120 (male, aged 32, served 8 years, discharged 2017) described that he was told by his prior chain of command he would be retained while on Christmas leave. However, the unit then failed to sign the paperwork to retain him. “*Arrived back at work the next year with a new Commanding Officer and Officer Commanding to be told I was facing termination.”* After appealing and escalating his termination several times, he reported being *“rejected and ejected. All because the last chain of command could not sign the paperwork as there wasn’t a Major in the unit at the time. Disgusting.”*

Some veterans who were medically discharged reported feeling so disempowered at discharge that they were coerced into signing benefit forms due to a desire to escape and/or uncertainty and anxiety about their future financial situation. For example, Participant 10 was made to sign compensation paperwork while admitted to the hospital for a minimal payout *“I was a medic who was medically discharged, it was horrible how hard I had to fight DVA (Department of Veteran Affairs) […]*”. After utilising advocates to revise his compensation he reported “*pension is ok but feal like get 75% of pay is a punishment I do not deserve, I gave my all, put life on the line, my illness led to divorce and separation from 2 young daughters, been a long time getting better and now am stable* 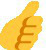
*”* Participant 10 (aged 53, served 15 years, discharged 2007).

Unlike the seemingly inferred transgressions from procedural neglect in subtheme 1, these more overt (rather than inferred) acts and omissions represent unambiguous transgressions of the mutual values and expectations held by the member, especially about their value and worth, and the way that the military was responsible for caring for them as people.

### Sub-theme 3 – bad actors and acutely harmful events

Many quotes within this theme were longer, more detailed, and depicted more acutely harmful events with longer-lasting effects, reflecting a profound sense of betrayal and loss of self. For example, Participant 189 (male, aged 42, served 3 years, discharged 2004), who was, days before his discharge date, deceived into signing paperwork he was told related to transportation of his possessions. Upon signing the paperwork, he was made to forfeit his military identification card and told he had been discharged.

*“[…]so I asked to get the duty driver to take me back to my place of work on the base and they said no you cant go back there you are now discharged, and made me walk down [street address] back to the lines where a truck had already come and taken all my stuff, I had no warning any of it was happening almost a week early, did not get to say my good byes or nothing, felt soulless as I walked down the road”* Participant 189 (male, aged 42, served 3 years, discharged 2004).

Participants in this category blamed a specific event or perpetrator for their negative discharge experience. Some veterans reported overt and intentional transgressions perpetrated by bad actors who abused power, insulted, or caused harm beyond what could be attributed as a necessary part of service requirements.

“*My discharge was a medical one due to [a] spinal injury that occurred in a training accident. I was made to do physical training and go on exercises with the injuries (ordered to get a clearance to attend and participate in all activities) that ultimately resulted in fusion surgery of the lumbar spine and chronic pain. Not likely to ever work again.”* Participant 45 (male, aged 42, served 4 years, discharged 2019).

*“My CO, coincidentally my supervisor, was easily one of the worst humans I have ever encountered. During my last week in uniform, my whole squadron was summoned for promotions and award of medals… Not a word was mentioned of my imminent departure after 30 + years of unblemished service and sacrifice. I found out afterwards, my CO forbade senior members of the squadron to attend my farewell. My CO made the transition period a nightmare.”* Participant 275 (male, aged 62, served 33 years, discharged 2022).

While bad actor experiences varied from petty and unnecessary insults at discharge to ongoing bullying and harassment, some instances were more severe, such as suffering sexual abuse and leaving the military to escape the perpetrator. For one veteran, an instance of abuse of power and attempted sexual exploitation of his partner, followed by a lack of institutional accountability, clearly represented a betrayal they could not endure and ultimately led to his discharge from the military.

*“I left because my RSM at the time demanded a blowjob from my wife in exchange for a promotion position (myself) the command did not investigate, look into this in any way”* Participant 278 (Male, aged 67, served 17 years, discharged 1991).

While some of the accounts contain clear harassment and bullying, the betrayal is not the bad actors alone but the way that the institution responds and a complete lack of treatment with principles of respect and justice. Some of these participants tended to view interactions and difficulties with the Department of Veteran Affairs (DVA) after discharge as further harm, viewing it as an extension of the harm suffered during their discharge.

*“My whole experience with being medically discharged was a joke. It’s a drawn out, degrading process and I thought leaving the ADF would solve all my problems… WRONG. DVA make it impossible to move on with your life, and their rehab providers for the return to work program are a bunch of self serving bullies.”* Participant 79 (Female, aged 33, served 6 years, discharged 2022).

*“I was admin discharged for 4 months after returning home from Afghanistan despite displaying symptoms of PTSD, MDD and alcohol use disorder. I reported the issues to the RMO, psychologist and Chain of Command at time of discharge. I had to go through retrospective discharge for support via Commonwealth Cuper Corporation. I received no help, referrals or anything after discharge date. I attempted suicide 3 times within a year of discharge.”* Participant 31 (male aged 33, served 5 years, discharged 2012).

One participant’s response emphasized the need for acknowledgment of wrongdoing as a step toward restorative justice. Without such acknowledgment, they questioned whether the military truly honors the efforts of its service members, reflecting a deeper disillusionment with the institution’s values and its commitment to fairness and accountability.

“*[…] I just wish Defence would write a letter one day and say ‘what we did was not what we would do today, we hope’. If not, we should stop believing that the military is there to fulfill our promises based on our efforts.”* Participant 95 (female aged 31, served 8 years, discharged 2022).

One veteran recounted severe and persistent misconduct by a superior over an extended period, where, despite a thorough investigation, the institution protected the perpetrator instead of the survivor.

*“I was medically discharged as a result of sexual harassment, bullying, sexual assaults & assaults (all from my supervisor over a 12 month period). The military police did a great investigation and wanted to charge him, but the ADF allowed him to leave to avoid charges. I feel utterly betrayed.”* Participant 108 (Female, aged 55, served 7 years, discharged 2023).

This example underscores the complexity of betrayal experiences, demonstrating that even when some aspects of the institution function correctly, individuals can still feel betrayed by the institution as a whole. It also demonstrates how cultural and structural aspects of military service, such as the chain of command structure, power imbalances, and prestige, increase the risk of abuse (Smith and Freyd, 2014). In the military, power imbalances and prestige are purposefully enacted through the chain of command rank structure and are necessary for the primary function of the military - warfighting. However, members elevated within this hierarchy have considerably more power and are considered literal ‘superiors’ relative to their ‘subordinates’. As such, a Brigadier, charged with the command of thousands of soldiers, can be seen to have considerably more value to the institution than a private. This high-status position within the hierarchy amplifies the potential for abuse, makes it difficult for victims to report abuse, and grants significant leverage for perpetrators to evade accountability. Perpetrators may manipulate their high status to shield themselves from repercussions, exploit institutional mechanisms to discredit and threaten victims or leverage the institution’s desire to maintain its reputation, prioritizing its image over justice for the victims (Royal Commission into Defence and Veteran Suicide, 2024c, pp. 151-154; Smith and Freyd, 2014).

While the severity of transgressions across these sub-themes varies, the underlying mechanisms remain consistent. Perceived transgressions during discharge can undermine the shared values that connect members to the group, posing a psychological threat to the individual’s social-moral identity and making them feel rejected and betrayed. This threat is amplified during the vulnerable period leading up to discharge, where veterans may become hypervigilant to signs of rejection, interpreting them as transgressions that conflict with their sense of belonging to the group. What constitutes a perceived transgression appears to be heterogeneous, varying for individuals, and dependent on various factors. For example, generally, members who had served for extended periods, perceived a high personal cost of their service or had a sense of closeness to the institution experience heightened betrayal when these are not recognized. Similarly, medical and involuntary discharge, when poorly processed and unsupported – heightens the experience of rejection and betrayal. Nonetheless, however large or small, transgressions during this period have the potential to be perceived by *some* veterans as significant betrayals with negative implications for belonging, self-esteem and self-concept long after discharge.

### Theme 2: Discharge as a loss of self

Theme 2 (Discharge as a Loss of Self) addresses research question 2 by showing how veterans perceive discharge experiences in relation to identity and belonging. It reveals veterans’ sense of liminality between military and civilian worlds and demonstrates betrayal when the military fails to fulfill transition support expectations. Those who most deeply invested in their military identity often felt greater betrayal, as the contrast between extensive recruitment/training support versus minimal discharge assistance violated their trust. This meaning-making process shows how veterans interpret institutional neglect as betrayal, compounding their identity disruption.

*“[…] That was the hardest part for a while, feeling so alone. Stuck in limbo, one foot in the military and the other in civilian life, unable to relate to either world at that moment and feeling so unsteady and unsure.”* Participant 90 (Female, aged 29, served 8 years, discharged 2022).

One participant explained in detail the processes of inculturation and identity formation that result in an overreliance on the institution and diminished non-military support networks, setting up members for a deep sense of loss at discharge.

“*People fail to realise that when you join the military, your three main support groups (family, friends, workmates) are merged into one, which creates an exaggerated dependency to the Army institution. After discharge, all of your pre-military support network have moved on or become distant, leaving you feeling lost, and needing to establish new networks and support structures. This becomes increasingly difficult with age.”* Participant 152 (aged 58, served 18 years, discharged 2017).

For some, the transition from what they ‘were’ to what they ‘are not’ was extreme and ongoing. One participant reported a sense of worthlessness after discharge, stemming from feeling devalued by civilian society which sharply contrasted with the cohesive ‘conformity’ valued in the military: *“On my own. No ‘support networks’ and no place available to me in civil society. Single, old, heterosexual, Caucasian. No value to anyone.”* Participant 190 (Male, aged 65, served 34 years, discharged 2018).

For many members, there was a clear expectation that the military would or should help them navigate reintegration and identity-related losses and difficulties. However, they found that no processes were in place to help them.

*“The military turned me into a machine. It took years. Then they discharged me without ANY ‘transition’ insights. They DIDN’T TURN US OFF!!!!!* 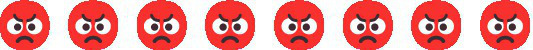
*The military stole my essence. It stole it because I ‘loaned’ it to them and they never gave it back […].”* Participant 43 (male, aged 61, served 21 years, discharged 1998).

Many members recounted the shock of the unexpected loss they felt the day on or after their discharge.

*[…] It felt surreal and I still did not want to believe I was done, I still do not most times. But I woke up the next day with nowhere to go, nothing to do, I no longer had a uniform, an identity, a place to go, a place to belong. I sat alone for hours wondering what the hell was my next move […].”* Participant 110 (male, aged 44, served 20 years, discharged 2022).

Despite mistreatment and, in some cases, abuse during their service, a minority of veterans expressed a strong desire to rejoin the military, highlighting the strength of their enduring attachment and longing for their past selves.

*“[…] I was in the prime of my life and being discharged and losing my working dog and leaving the one career that I had planned on having my entire life did leave me with a massive loss of self and belonging. But after all the mistreatment the Army is so ingrained in my sense of self that I have tried and would rejoin in an instant.”* Participant 109 (male, aged 49, served 10 years, discharged 2003).

Yet, for one individual who did not strongly identify with the military, the discharge process was a less significant event.

*“I was a Civilian who wore a Particular Set of Clothing that I went to work in. For me the Australian Army was a “Job” like any other. When I discharged I found another “Job” and got on with my life. The AABC [Australian Army Band Corps] Members who wanted a “Career,” well they were Used, Abused and Mistreated.”* Participant 116 (male aged 68, served 11 years, discharged 1981).

These examples highlight that individuals with a less centralized military identity may fare better in transition due to experiencing a reduced identity loss – both through the military identity being less centralized (taking up less of one’s self-concept) and having a civilian job role equivalent aiding self-continuity (Binks and Cambridge, 2017; Grant et al., 2025). Conversely, those with a highly centralized military identity who are deeply invested in the military may be at greater risk of abuse precisely because of their significant emotional and psychological investment in the institution. This is in line with research demonstrating that members in combat roles without civilian job equivalents often have a more centralized identity and difficult transition (Binks and Cambridge, 2017).

Participant 162 (male, aged 48, served 29 years, discharged in 2022) described discharge as a brutal moment of loss and disconnection from the veteran’s previous way of life, especially for members who are involuntarily discharged. He noted that practical restrictions worsen these losses in the Australian military context:

*“[…] From the moment their ID card is handed in, members can no longer access even non-technical areas of defence establishments such as canteens, wet-messes, bars, gyms, sporting facilities, or recreation areas. This contrasts with the US armed forces where veterans may continue to access non-technical areas of military bases by using their ‘retired veteran’ ID card. […].”* Participant 162 (male, aged 48, served 29 years, discharged 2022).

Indeed, such a definitive removal from military bases is not only symbolic of the member’s separation from the group and institution but also creates practical barriers to maintaining existing social connections. For some veterans, especially those who lived on base, the majority of their social connections were developed and maintained on military bases. Many social interactions that support a sense of belonging and help maintain psychosocial functioning are incidental, occurring on base at the gym, pool, mess, or bars. For members with a highly centralized identity or a strong sense of connection to the military community, access to such non-technical areas of bases may facilitate these spontaneous interactions, potentially minimizing losses and supporting psychological well-being. Access would additionally serve as a symbolic but meaningful act, demonstrating to members they are still part of the military family.

### Theme 3: Negative discharge experiences as negative centralizing events and ‘stuck-points’

This theme addresses research questions 2 and 3 by exploring how veterans may internalize negative discharge experiences as defining life events. The theme suggests these experiences potentially function as “stuck points” that could hinder adjustment to civilian life by fostering maladaptive beliefs about safety, trust, power, and self-worth.

Across the participants’ stories, we observed many similarities in how they narrated their discharge experiences, akin to individuals experiencing similar symptoms in other traumatic contexts. As researchers and clinicians working with “stuck points”—maladaptive beliefs that arise in response to traumatic experiences—we noticed that these narratives frequently revolve around themes such as safety, trust, power/control, and self-esteem (Resick et al., 2017). Related and overlapping with Themes 1 and 2, many veterans described their negative discharge experiences in great detail, recounting ongoing repercussions on their sense of self, worth, belonging, and well-being long after discharge. For some participants, these events appear to have become negative centralizing life events and/or ‘stuck points’ that hinder their adjustment to civilian life.

Research on autobiographical recall indicates that significant life transitions, particularly those involving profound changes to life circumstances, enhance the salience and emotional intensity of events proximal to the transition. These events are more readily recalled, both voluntarily and involuntarily, compared to more mundane life experiences (Enz and Talarico, 2016; Graber and BrooksGunn, 1996; Mobbs and Bonanno, 2018; Rubin et al., 2008). These highly distinctive and accessible memories serve as reference points that organize memories of less significant events (Fitzgerald et al., 2016; Rubin et al., 2008). As such, these pivotal events and the meanings ascribed to them typically structure one’s life narrative, shaping self-concept and providing a coherent cause-and-effect account of their present circumstances and state of being (Berntsen and Rubin, 2006; McAdams and McLean, 2013; Pillemer, 2001).

However, when these life-stage events are significantly adverse and associated with negative emotions, they can become a central focus in an individual’s narrative – a negative centralizing event (Berntsen and Rubin, 2006; Fitzgerald et al., 2016). Negative centralizing events are adverse experiences that dominate one’s life story, becoming part of the individual’s identity and a focal point around which other memories and beliefs are organized. The negative emotions tied to these events can distort perceptions of self, others, and the world, leading to a pervasive negative outlook. This shift can cause individuals to interpret otherwise neutral or positive events more negatively (Boelen, 2009).

Furthermore, significant negative or traumatic experiences can challenge one’s core beliefs and understanding of the world as fair, predictable, and safe. Individuals seek to comprehend life events through cause and effect to maintain a sense of control and predictability (Resick et al., 2017). When they encounter events that contradict their core beliefs, they may develop cognitive distortions to assimilate these events according to their prior understanding, rather than integrating new evidence that the world can be unjust or unpredictable (Resick et al., 2017). This can lead to the development of overly simplistic or extreme, rigid, and overgeneralized maladaptive beliefs—referred to as ‘stuck points’—which hinder recovery. For example; Participant 259 (male, age 56, served 24 years, discharged 2012 – previously quoted in Theme 1).

*“[…] I’ve had nothing to do with the military since. I still cannot bring myself to go to ANZAC day…. I regret my 24 years of service. I regret the damage that my time in service that is still affecting my life and family. I’m not in the military any more but I’m still not comfortable to civilian live. I just do not fit in. I am broken.”*

In this case, his potential stuck points, “I just do not fit in” and “I am broken,” have significant implications. The self-perception of being “broken” reflects cognitive distortions like overgeneralization—assuming that because he struggles in certain areas, he is fundamentally flawed everywhere—and labeling, where he defines his entire identity by his perceived shortcomings. Such distortions prevent him from recognizing his strengths and potential for growth. The belief that he does not fit in has driven him to avoid military-related events like ANZAC Day, reinforcing a pervasive sense of alienation from both his military past and civilian present. These stuck points perpetuate his sense of disconnection, preventing him from finding a sense of belonging, certainty, worth, and purpose in his post-service life. As a result, these entrenched beliefs likely keep him trapped in a cycle of negative emotional states and avoidance, hindering his adjustment to civilian life. See also Participant 131.

*“Its like a jigsaw puzzle, the Army didnt put the pieces back in the places where they were before enlistment. I have lost my identity. I know nothing else and I have been trying to fill the void it has left in my life, everything I have tried since discharge over 30 years ago ie volunteer work etc has failed. Life now has little meaning….*” Participant 131 (male, aged 67, served 17 years, discharged 1991).

His belief that “the Army did not put the pieces back,” reveals a deep-seated reliance on the military to restore his identity after service. This reliance acts as a ‘stuck point’, fostering a perceived dependency on the institution and fuelling feelings of powerlessness. His identity remains anchored to the military’s actions—or inactions—rather than his own capacity for adaptation and growth. This dependency renders the task of redefining his identity seemingly unattainable, reinforcing his conviction that no other roles or pursuits can ‘fill the void’ left by his former military identity.

## Discussion

### The impact of negative discharge experiences and betrayals

This study sought to identify specific aspects of military discharge experiences that contribute to the vulnerabilities associated with this period, such as loss of identity, purpose, and belonging, and to understand why feelings of betrayal arise during these times. Our analysis suggests that negative discharge experiences most often occurred when veterans perceived the discharge or experiences surrounding it, as transgressions by the military institution or members within it. Perceived transgressions varied, ranging from seemingly minor, such as a sense of disregard from their unit, to more significant, involving direct rejection from the military due to service-related harm or overt experiences of betrayal. The consequences of these transgressions did not scale proportionally with their severity; both minor and more severe transgressions could lead to profound, long-lasting negative impacts for some veterans.

Our analysis revealed that sub-theme 2, “Left Harmed and Rejected,” appeared more frequently in accounts from medically or involuntarily discharged veterans. This pattern may stem from these veterans’ unique circumstances. Medical discharge typically follows service-related injuries or conditions, potentially creating a perception of double harm: first, in sustaining an injury during service, and second, in being devalued and/or separated due to that injury. Some veterans may interpret this sequence as having sacrificed their health for the institution, only to then face separation because of that sacrifice. The non-voluntary nature of their discharge might contribute to feelings of powerlessness, differing from the experiences of those who chose to leave. Furthermore, medically discharged veterans often navigate extended discharge processes with medical boards, administrative procedures, and compensation claims—a journey that can include periods where veterans remain physically present in military environments while feeling increasingly disconnected, possibly intensifying sensations of rejection.

We acknowledge that many of the experiences in participants’ accounts share conceptual overlaps with moral injury; however, we emphasize key distinctions and have not used this terminology for two reasons. Firstly, none of the participants used language that directly addressed or spoke to experiences of moral injury. Secondly, because we see a danger of pathologizing and individualizing these experiences. Moral injury is not uniformly defined, but is commonly characterized as “*an act of transgression that creates dissonance and conflict because it violates assumptions and beliefs about right and wrong and personal goodness […] resulting in psycho-bio-social impairment characterized by diminished opportunity for ‘life affirmation’*” (Litz et al., 2024, p.698). Current models of moral injury have been critiqued for their narrow, individual-centered, belief-focused perspectives, which tend to reduce morality to static beliefs or moral rules and overlook relational and social contexts that are essential to moral experience (Acampora et al., 2024). We used the linked phrase social-moral to underpin the idea that what is “injured” in these transgressions is the underlying sense that one is included and respected as a good and appropriate group member or relationship partner (Woodyatt et al., 2022), not the morality of any specific actions. Moral Injury frameworks often center on internal condemnation and pathologize individual responses while neglecting broader relational and contextual factors (Acampora et al., 2024; Frankfurt and Frazier, 2016). Moreover, viewing moral injury solely through a lens of trauma or turning moral injury into another label for a type of psychopathology constrains the concept to discrete “immoral events,” limiting its applicability to sustained, systematic, and cumulative psycho-social harm over time, which contrasts with our findings of ongoing harm during protracted discharges (Acampora et al., 2024; Boudreau, 2011).

In contrast, our proposed mechanisms shift the focus from the individual to external factors—including social, relational, and institutional influences—that shape harm arising from negative discharge experiences. These mechanisms account for both cumulative harm over time and discrete events, thus addressing the underlying transgressions and betrayals at discharge. Rather than framing these harms as a mere violation of personal beliefs, we conceptualize them as rooted in disruptions within moral relationships and broader social contexts.

We argue that a key mechanism of harm lies in how perceived transgressions undermine the shared values connecting members to the military institution. Violation of these shared values severs the individual’s connection to the military and their military self (from which they often primarily derive their self-concept), posing a psychological threat to their social-moral identity and fostering feelings of rejection, betrayal and diminished social-moral identity. Such transgressions from a close-knit and trusted group that was expected to support its members destabilize veterans’ sense of identity, creating uncertainty about their place in (and understanding of) the world, their purpose, future and self-worth. The extent of this perception significantly influences the likelihood of negative psychological outcomes.

In the context of military service, characterized by a highly centralized identity, quasi-familial bonds, loyalty, and an intense reliance on trust and perceived safety, negative discharge experiences (especially when interpreted as betrayals) may constitute a trauma-like event because of the fundamental importance of our attachment bonds as humans for feelings of safety. From a psychosocial needs perspective, our brain interprets these threats as survival-related threats. The relationship between social threat and psychological responses is well-founded [for review see Baumeister and Leary (1995) and Haslam et al. (2021)]. For example, social rejection activates neural pathways associated with physical pain (Eisenberger, 2012) and triggers stress responses that elevate cortisol levels and inflammation, adversely affecting health (Slavich and Irwin, 2014). Further, chronic social stressors can lead to epigenetic changes that dysregulate stress-response systems, increasing vulnerability to mental health disorders (Cole, 2014; Szyf et al., 2008).

While direct betrayals by bad actors and intensely negative events can intensify social threat and make discharge more likely to be a trauma-like event with trauma-like symptoms, a key finding of this study was that harm was also perpetrated through neglect in the mundane – not just through explicit harm. Despite many of our participants lacking PTSD diagnostic Criterion A events, which involves exposure to actual or threatened death, serious injury, or sexual violence (American Psychiatric Association, 2013). The rejection and betrayal they experienced appear to inflict a wound to the very core of the individual’s identity and sense of psychological safety and trust. And this communicates negative messages about their worth—for if they were truly valued, how could such a trusted and loyal institution betray them so profoundly? It is unsurprising, then, that significant experiences of institutional betrayal can mirror betrayal trauma and create/exacerbate existing PTSD symptoms despite the betrayal lacking PTSD diagnostic Criterion A events (Christl et al., 2024; Smith and Freyd, 2013, 2014).

Our proposed mechanisms shift focus from the individual to external factors—particularly social, relational, shared values, and institutional influences—that shape the harm stemming from negative discharge experiences. Rather than framing moral injury solely as a violation of personal beliefs, we conceptualize it as rooted in disruptions within moral relationships and social contexts.

Instead of focusing on the semantics of categorizing these discharge experiences within moral injury or seeking the simplest available theory, the essential question is: “Where’s the root cause of harm?” The harm is fundamentally rooted in the perceived transgression that violated shared values, destabilizing the social-moral identity of the veteran. This rupture disconnects veterans from their military peers and prior military identity, making it difficult to envision or develop a future sense of self that is valued and socially connected. Consequently, the normative process of transitioning—re-evaluating one’s identity—becomes fraught with uncertainty, complicating foundational questions: Who am I now? How should I behave? What should I believe? Who will I become?

In sum, military discharge is a known period primed for significant psychological vulnerability. Our findings suggest that the quality of the discharge experience may be the difference between a healthy integration of the transition into the life narrative or the development of a negative centralizing event and possible stuck points. The latter being associated with an increased risk of developing a globally negative view, low self-esteem and self-concept, rumination, worries, increased memory intrusions of the event, and avoidance behaviors hampering the veteran’s ability to transition effectively (Berntsen and Rubin, 2006; Boelen, 2009; Fitzgerald et al., 2016).

### Discharge as a psychosocial hazard

The findings of this paper suggest it is not the act of discharge itself but the interpersonal interactions and perceived fairness, injustice, and neglect that are a risk of harm during discharge. Addressing discharge experience issues requires a shift from treating individual symptoms to tackling the systemic factors perpetuating these harmful experiences. To effectively address these institutional problems, we must incorporate organizational psychology perspectives, viewing these issues not just as individual or interpersonal problems but as matters of organizational process and justice. There is a need to ensure discharge processes are conducted with the utmost fairness, transparency, consistency, and respect to reduce instances of negative discharge experiences.

Further, we must consider the psychosocial hazards [aspects of work with the potential to cause psychological harm; Safe Work Australia (2024)] linked to military indoctrination practices that centralize a member’s military identity, making them vulnerable to abuse, betrayal, (Smith and Freyd, 2014) and identity loss upon discharge and precipitating integration difficulties (Royal Commission into Defence and Veteran Suicide, 2024a. pp 13, 243-244, 269). The organizational psychology perspective raises important questions: To what extent are these practices and the resulting centralized military identity necessary for military cohesion and operational effectiveness? Can these goals be achieved without centralizing a military member’s identity? How can these risks be reduced? Exploring these questions allows us to evaluate whether the negative consequences of indoctrination practices are necessary, reasonable, and practicable within military service. If they are not, minimizing identity centralization would reduce identity and transition-related difficulties. However, if they are deemed essential, then military institutions have a moral and legal responsibility to minimize this workplace psychosocial hazard and address identity-related issues and their negative impacts on discharging members.

### Improving the discharge experience

Despite the commonalities and conformity inherent in military service, members’ experiences and needs at discharge are uniquely diverse. What is important for some veterans may not hold the same significance for others. Therefore, any meaningful attempt to improve discharge experiences requires a co-designed approach that places veterans with lived experience at the centre. Implementing a co-designed approach informed by the lived experiences of service members is necessary, given the diversity and heterogeneity of military experiences and subcultures. Utilizing established methodologies like human-centered design processes can ensure that changes are meaningful, structured, effective, and continually improved. Such approaches, which involve stakeholders in designing processes that affect them, have been shown to provide consumers with a sense of autonomy and have more effective and acceptable outcomes (Brown and Wyatt, 2010; Sanders and Stappers, 2008).

### Limitations and future research

This study has several limitations that should be acknowledged. The use of online recruitment methods may have introduced self-selection bias, with individuals who had strong negative experiences potentially being more motivated to participate. Additionally, our reliance on open-text survey responses from a primarily quantitative study limited opportunities for probing and clarification that would be available in interview-based qualitative research. While this approach allowed us to analyze perspectives from a diverse sample spanning nearly six decades of service, it potentially restricted the contextual depth of individual narratives. The sample was also older on average, which may limit the generalizability of our findings to younger or contemporary veterans, or those with neutral or positive discharge experiences. However, it is noteworthy that many of the most impactful examples cited in this paper came from veterans who had recently been discharged. And importantly, amongst those with a considerable amount of time since discharge, the harm can still remain. Participants’ accounts of their discharge experiences may also be influenced by autobiographical recall biases, particularly for veterans who were discharged some time ago. However, if the provided accounts had become more negative over time, this might reinforce our notion that negative discharge experiences can become negative centralized events.

Given that our findings indicate that medically discharged veterans are particularly vulnerable to negative discharge experiences, transgressions, and betrayals—and with an increasing trend of Australian Veteran medically discharging, now approximately two--thirds (Royal Commission into Defence and Veteran Suicide, 2024b, p. 389)—it is crucial to further investigate these findings within this specific population.

Intensive longitudinal research is needed to further explore and validate the findings of this study. Such an approach would gather prospective data on members’ experiences before, during, and after discharge, potentially utilizing momentary assessment and experience sampling methods. There are existing measures for constructs such as event centrality, identity loss, and institutional betrayal. A large-scale study involving veterans from nations across the Five Eyes alliance nations would provide crucial sequential and temporal insights into many of the concepts discussed in this paper.

## Conclusion

The findings of this study support our prior research and align with testimonials from the Royal Commission into Defence and Veteran Suicide, both of which link negative discharge experiences to institutional betrayal, identity loss, and mental health challenges (Grant et al., 2025; Royal Commission into Defence and Veteran Suicide, 2024a. pp 13, 31-43, 243-244, 269). This study extends these insights by highlighting specific mechanisms that may contribute to making military discharge a particularly vulnerable period. Specifically, it describes mechanisms through which negative discharge experiences may foster perceptions of transgressions and betrayals by the military institution, violating previously mutually shared values, threatening moral-social identity, and severing their psychological bonds with the military.

The consequences of these perceived transgressions appeared subjective and did not necessarily scale with their objective severity. Both minor and severe transgressions had potential to lead to a profound sense of betrayal and long-lasting negative impacts for some veterans. The psychological consequences of these transgressions seem to overlap with known factors and issues among discharging veterans, including identity loss, loss of purpose, social isolation, and worthlessness (Royal Commission into Defence and Veteran Suicide, 2024a, pp. 56–60).

Moreover, for some veterans who perceived significant institutional betrayal, the incongruence between their expectations and their actual discharge experience seemed to result in the development of a negative centralized event—where the discharge experience became a focal point of their negative worldview. This centralization appeared to contribute to the development of distorted cognitions or ‘stuck points’ hindering transition—concepts that are observed in individuals managing trauma-related responses. These findings align with existing research demonstrating that institutional betrayal can mirror betrayal trauma and create or exacerbate PTSD-like symptoms.

## Data Availability

The datasets presented in this article are not readily available because of ethical restrictions. Requests to access the datasets should be directed to cam.grant@flinders.edu.au.
